# Pro-Apoptotic Activity of *Epi*-Obtusane against Cervical Cancer: Nano Formulation, In Silico Molecular Docking, and Pharmacological Network Analysis

**DOI:** 10.3390/ph16111578

**Published:** 2023-11-08

**Authors:** Omnia Hesham Abdelhafez, Islam M. Abdel-Rahman, Eman Alaaeldin, Hesham Refaat, Refat El-Sayed, Sami A. Al-Harbi, Ahmed M. Shawky, Mohamed-Elamir F. Hegazy, Alaa Y. Moustafa, Nourhan Hisham Shady

**Affiliations:** 1 Department of Pharmacognosy, Faculty of Pharmacy, Deraya University, Universities Zone, New Minia 61111, Egypt; 2 Department of Pharmaceutical Chemistry, Faculty of Pharmacy, Deraya University, New-Minia 61111, Egypt; dr.islam_moh@deraya.edu.eg; 3 Department of Pharmaceutics, Faculty of Pharmacy, Minia University, Minia 61519, Egypt; eman.alaa@deraya.edu.eg; 4 Department of Pharmaceutics, Faculty of Pharmacy, Deraya University, Universities Zone, New Minia 61111, Egypt; 5 Department of Pharmaceutical Sciences and Experimental Therapeutics, College of Pharmacy, University of Iowa, Iowa City, IA 52246, USA; hesham-abdelsamad@uiowa.edu; 6 Department of Chemistry, University College in Al-Jamoum, Umm Al-Qura University, Makkah 24231, Saudi Arabia; reabdelfattah@uqu.edu.sa (R.E.-S.); sadharbi@uqu.edu.sa (S.A.A.-H.); 7 Chemistry Department, Faculty of Science, Benha University, Benha 13518, Egypt; 8 Science and Technology Unit (STU), Umm Al-Qura University, Makkah 21955, Saudi Arabia; amesmail@uqu.edu.sa; 9 Chemistry of Medicinal Plants Department, National Research Centre, El-Tahrir Street, Dokki, Giza 12622, Egypt; elamir77@yahoo.com; 10 Zoology Department, Faculty of Science, Sohag University, Sohag 82524, Egypt; alaa.moustafa@science.sohag.edu.eg

**Keywords:** *aplysia*, *epi*-obtusane, cervical cancer, pharmacological network, liposomes

## Abstract

Cancer is a major disease that threatens human health all over the world. Intervention and prevention in premalignant processes are successful ways to prevent cancer from striking. On the other hand, the marine ecosystem is a treasure storehouse of promising bioactive metabolites. The use of such marine products can be optimized by selecting a suitable nanocarrier. Therefore, *epi*-obtusane, previously isolated from *Aplysia oculifera*, was investigated for its potential anticancer effects toward cervical cancer through a series of in vitro assays in HeLa cells using the MTT assay method. Additionally, the sesquiterpene was encapsulated within a liposomal formulation (size = 130.8 ± 50.3, PDI = 0.462, zeta potential −12.3 ± 2.3), and the antiproliferative potential of *epi*-obtusane was investigated against the human cervical cancer cell line HeLa before and after encapsulation with liposomes. *Epi*-obtusane exhibited a potent effect against the HeLa cell line, while the formulated molecule with liposomes increased the in vitro antiproliferative activity. Additionally, cell cycle arrest analysis, as well as the apoptosis assay, performed via FITC-Annexin-V/propidium iodide double staining (flow cytofluorimetry), were carried out. The pharmacological network enabled us to deliver further insights into the mechanism of *epi*-obtusane, suggesting that STAT3 might be targeted by the compound. Moreover, molecular docking showed a comparable binding score of the isolated compound towards the STAT3 SH2 domain. The targets possess an anticancer effect through the endometrial cancer pathway, regulation of DNA templated transcription, and nitric oxide synthase, as mentioned by the KEGG and ShinyGo 7.1 databases.

## 1. Introduction

Cancer is the second-leading causative agent of death worldwide after cardiovascular diseases [1]. Each year, there are more than 10 million new cancer cases with over 6 million associated deaths, approximately accounting for 12% of worldwide mortality [2]. A WHO report announced that the cancer-associated death rate will reach 12 million cases by 2030. In addition, an American Cancer Society report showed that nearly 27 million patients are expected to have cancer, with a probable increase in the mortality rate by 17.5 million deaths by 2050 [3,4,5]. While cancer causes nearly 13% of all deaths in the world, more than 30% cancer-related deaths could be avoided by changing or evading its main triggers [6]. Consumption of tobacco and alcohol, harmful food habits, lack of exercise, and dangerous radiation are some of the risk factors for the disease [7,8].

Additionally, human cervical cancer is considered the fourth, most lethal cancer type among women in developed and developing countries [9]. Moreover, the newly reported cases of cervical cancer among women are 3.2% worldwide according to a World Health Organization report [10]. Cervical cancer therapy includes several scenarios, including chemotherapy and radiation therapy [11]. Unfortunately, these conventional methods pose many drawbacks that may hinder their clinical application in some cases. Moreover, they affect the quality of life in cancer patients in a negative manner due to their side effects [12].

Natural products have been implicated in the treatment of different diseases due to the powerful effect of their components in curing several human illnesses [13,14,15]. Additionally, they have numerous unique scaffolds, which afford more and more inspiration for drug discovery [16,17,18,19,20,21,22,23,24,25]. Though terrestrial plants as well as bacteria have usually been the main resources of natural metabolites, in the last 30 years, marine ecosystems, including their invertebrates, plants, as well as bacteria, have also been the main sources of numerous novel structural metabolites, which are identified as natural marine products [26,27,28,29,30,31,32].

Additionally, more than 70% of our planet is occupied by oceans, and life on Earth derives from the oceans [33]. Among different marine organisms, sea hares ranked at the top as a storehouse of new bioactive natural metabolites [34]. *Aplysia* among other sea hares is the most studied genus by scientists as a rich resource of secondary molecules, mostly of dietary origin [35]. Moreover, a huge number of secondary molecules obtained from the genus *Aplysia* were identified as halogenated terpenes; nevertheless, these animals are known as a resource of metabolites from different chemical scaffolds, such as macrolides, alkaloids, and sterols, which showed cytotoxic, antiviral, antifungal, antibacterial, and/or antifeedant potential [36]. Unfortunately, the optimum use of the therapeutic effects of such marine natural products has been limited due to the scarce isolated amount from the original source, insufficient stability and solubility in physiological fluids, and low targeting [37]. Formulation of extracted marine products within nanocarriers may be a promising strategy to overcome the obstacles related to those promising therapeutic cargo [38,39,40,41,42]. Liposomes are versatile lipid-based nanocarriers that are able to entrap both the hydrophilic as well as lipophilic constituents of natural extracts [43]. They have the advantages of being biocompatible and achieving site-specific targeting [44]. In this study, the potential biological activity of *epi*-obtusane was screened to reveal its antiproliferative activity toward cervical cancer; subsequently, a formulation study was carried out for the entrapment of the *epi*-obtusane within a suitable liposomal formulation, which was evaluated in terms of enhancement of targeted delivery and cellular uptake of the entrapped constituents. Furthermore, the antiproliferative potential of *epi*-obtusane was investigated against human cervical cancer cell line HeLa cells before and after encapsulation with liposomes. Regarding the mechanism of action for its anticancer activity, a pharmacological network was constructed, displaying that *epi*-obtusane may act as a STAT3 inhibitor in its anticancer activity; this activity was evaluated through in vitro analysis and in silico docking, which was followed by gene ontology and enrichment analysis.

## 2. Results and Discussion

### 2.1. In Silico-Based Determination of the Bioactivity

**Figure 1:**
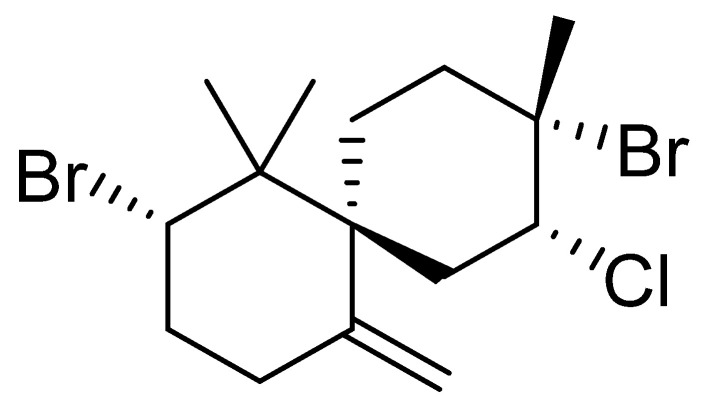
Structure of *epi*-obtusane.

Biological activity predictions with the aid of software have become a crucial preliminary step in the drug discovery workflow. Such in silico-based experiments are valuable for exploring natural products for new bioactive metabolite [45]. Consequently, the structure of *epi*-obtusane (Figure 1) was submitted to the neural network-based prediction software (PASS) to putatively determine the potential biological activity of *epi*-obtusane. This software evaluates the structural similarity of a massive number of inhibitors recorded for numerous molecular targets. Results revealed a high possibility of antineoplastic activity for the investigated compound with Pa of 0.970. This is consistent with a previous study that revealed cytotoxic activity for the same compound with Pa of 0.973 as an antineoplastic agent, particularly toward cervical cancer [46].

### 2.2. Epi-Obtusane-Containing Liposomes

**Figure 2:**
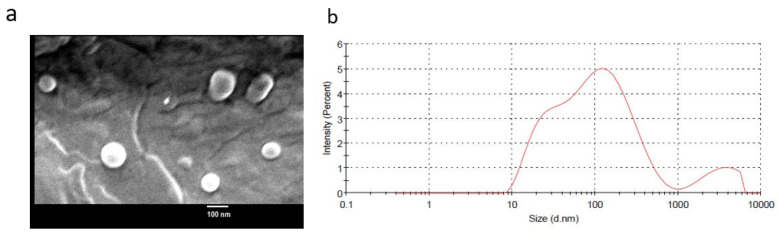
(**a**) SEM and (**b**) particle size distribution of *epi*- obtusane-containing liposomes.

Liposomes of *epi*-obtusane were successfully prepared with a small particle size and homogenous distribution (size = 130.8 ± 50.3, PDI = 0.462) (Figure 2). The vesicles have a zeta potential of −12.3 ± 2.3.

### 2.3. Antiproliferative Activity of Epi-Obtusane and Its Liposomal Formulation

**Figure 3:**
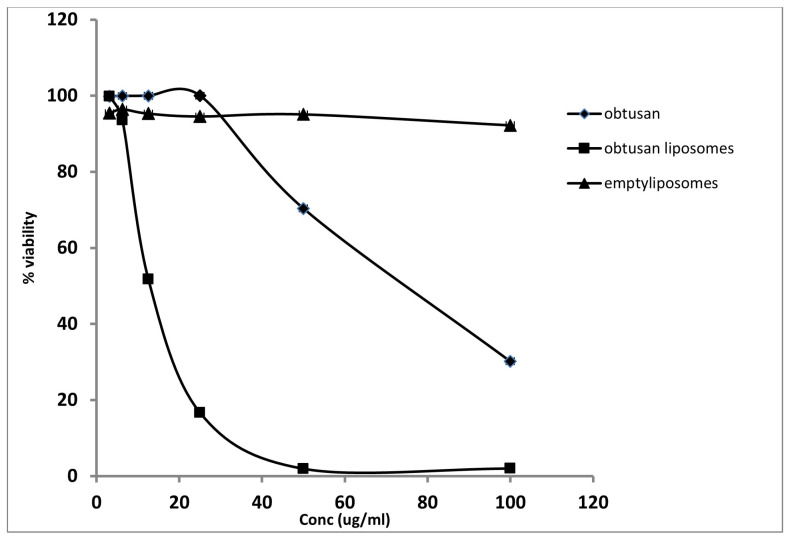
Anti-proliferative activity of *epi*-obtusane, *epi*-obtusane liposomes and empty liposomes against the HeLa cell line.

To test the potential of liposomal encapsulation on the enhancement of the delivery of the entrapped cargo, the *epi*-obtusane-containing liposome was in vitro screened for its anti-proliferative activity towards the HeLa cell line. Results showed *epi*-obtusane inhibited the growth of the HeLa cell line with an IC_50_ value of 76.8 ± 2.3 µg/mL (Figure 3). Entrapment of the molecule within the formulated liposomes enhanced its cytotoxic effect against the tested cells. IC_50_ of the encapsulated molecule was minimized for the HeLa cell line to 15.65 µg/mL (*p* < 0.001), (Figure 3). Doxorubicin, as a reference drug, showed an IC_50_ value of 12.39 ± 0.9 µg/mL. Moreover, the effect of *epi*-obtusane, *epi*-obtusane-containing liposome and empty liposomes on non-cancer cervical cell line (PCS-480-011) was studied in comparison to the reference drug doxorubicin. Results show that IC_50_ values were 78.79 ± 1.2, 355.35 ± 12.8, 126.53 ± 11.5, 34.69 ± 5.3, respectively. It is obvious that formulated liposomes of *epi*-obtusane have exerted a safer effect on the normal cell than doxorubicin (*p* < 0.001). This selective effect of *epi*-obtusane-containing liposome to tumor cells may be attributed to the well-known enhanced permeation retention (EPR) effect of liposomes. Liposomes target tumor tissue selectively due to the unique structure of the tumor tissue, including high vascularity and permeability of cells [47,48].

This is compatible with former studies that reported the effect of nano-carriers enhancing the cellular uptake and accessibility of the entrapped cargo [49,50]. The small nanosized carriers assist cellular entry and uptake by minimizing the energy necessary for endocytosis [51].

Despite the promising cytotoxic effect of *epi*-obtusane against the tested cell line, poor solubility and delivery hinders the best use of the molecule. Entrapment of *epi*-obtusane within the formulated liposomal dosage form enhances the solubilization and cellular internalization of *epi*-obtusane through the permeable vasculature of the tumor tissue. Moreover, the potential of nano-liposomes to entrap both the hydrophilic and lipophilic constituents of the molecule maximizes its therapeutic effect. In addition, cholesterol, one of the constituents of the bilayer membrane, improves the cellular uptake of liposomes-containing *epi*-obtusane. In addition, encapsulating *epi*-obtusane within a suitable nanocarrier would enhance the targetability of the entrapped cargo to the tumor tissue, either spontaneously via EPR effect or by a different mechanism such as using pH sensitive polymers. In other words, encapsulating such an anti-proliferative payload into a suitable liposomal formulation would enhance its cellular uptake, maximize the cytotoxic potential of such a promising molecule, reduce the dose required and reduce the side effects on the normal cells.

### 2.4. Cell Cycle Analysis on HeLa Cell Line Treated with Epi-Obtusane

**Figure 4:**
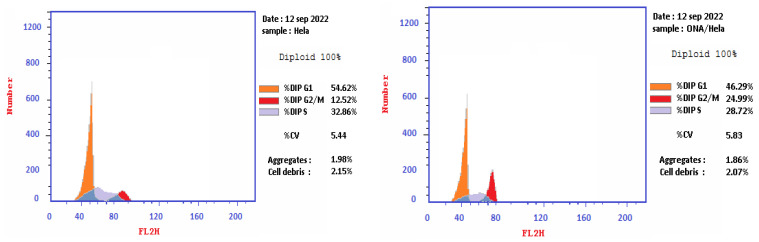
Effect of *epi*-obtusane on DNA-ploidy flow cytometric analysis of HeLa cells after 24 h.

Cell cycle analysis was performed to determine whether the observed reduction in cellular viability, caused by *epi*-obtusane, could be linked to alterations in the cell cycle. One of the fundamental biological responses to safeguard genomic integrity in the face of DNA damage involves the imposition of cell cycle arrest. Following DNA damage, cell cycle arrest can manifest at specific stages, including G1, the S phase, or prior to mitosis at the G2/M checkpoint. G2/M arrest, also known as G2/M checkpoint arrest, is a critical regulatory mechanism in the cell cycle that ensures proper DNA replication and integrity before a cell proceeds to the mitotic (M) phase. Several factors or events can trigger G2/M arrest, such as DNA damage, chromosomal abnormalities, DNA cross-links and adducts, and the activation of tumor suppressor proteins; the results showed that *epi*-obtusane increased the frequency of arrested cells at the G2/M phase, and, simultaneously, the cells population in the G1 and S phases declined. It showed a significant increase in the percentage of cells at the G2/M phases with 24.99%, sequentially, compared to control with 12.52% (Table 1, Figure 4). This evidence suggested that the *epi*-obtusane mediated antitumor activity was mediated through G2/M phase cell cycle arrest and apoptosis [52,53].

### 2.5. Apoptosis Determination by Annexin-V Assay

**Figure 5:**
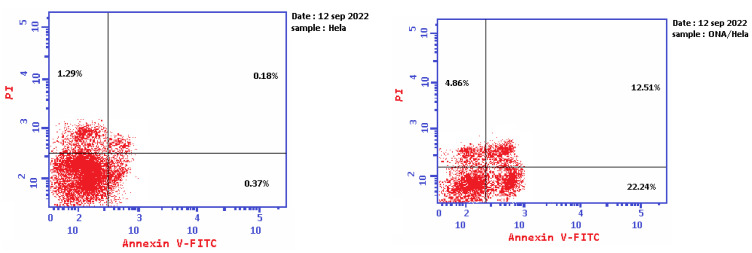
Representative dot plots of HeLa cells treated with *epi*-obtusane for 24 h and analyzed by flow cytometry after double staining of the cells with annexin-V FITC and PI.

To certify the capability of *epi*-obtusane to induce apoptosis, a biparametric flowcytometric analysis was executed. Two stains were used, one was propidium iodide, which can enter the dead cell, interact with its DNA, and stain the protein annexin-V. This protein is attached to the expressed phosphorylated serin outside the cell membrane of the apoptotic cells and stains it with marked green fluorescence. This double staining could differentiate between the different types of apoptosis in the tested cells at early and late apoptosis, and necrosis that may occur after 24 h of incubation with the selected apoptotic agent. As shown in Figure 5 and Table 2, after the incubation of HeLa cells with *epi*-obtusane at its IC_50_ concentration, a decline in the survival percentage of the cells was recorded, accompanied by elevations in the annexin-V-stained cells (lower right square) that were 60 times higher than the control, indicating the occurrence of an early apoptotic effect. Furthermore, intensive staining of the tested cells with both annexin-V and propidium iodide resulted in 70 fold increases compared to the control, which indicates a late apoptotic effect of the tested compound (upper right square). While there was a slight increase in necrotic cell numbers (4.86) due to treatment with *epi*-obtusane, a significant increase in the number of the apoptotic cells occurred (39.61). These results displayed that apoptosis is a probable mechanism by which *epi*-obtusane may act in cervical cancer.

### 2.6. Pharmacological Network

#### 2.6.1. Collection of Potential Targets for Antitumor Activity

In order to obtain the mechanism of action that *epi*-obtusane may have encountered in its antitumor activity towards cervical cancer, a protein–protein interaction network was constructed. The proposed approach starts with the selection of a list of proteins, known to be encountered in cervical cancer, collected from the Gene Cards database (https://www.genecards.org/ (accessed on 1 February 2023)) [53]. A total of 7767 target genes were retrieved from the database. Almost 6759 genes are related to protein coding. The maximum Boolean model score was 213, the minimum score was 0.126 and the median was 3.03. The Gene Cards Inferred Functionality Scores (GIFts) predicted the degree of gene functionality with a maximum score of 59, minimum score of 4, and median of 44. In total, 2079 target protein coding genes with a relevance score greater than 3.03 and GIFts greater than 44 were retrieved [54].

#### 2.6.2. Construction of Protein-Protein Interaction (PPI) Network

The collected proteins were submitted to the STRING application (https://string-db.org/ (accessed on 1 February 2023)) [19] for protein–protein interaction (PPI) analysis, selecting “Homo sapiens” as the type of species, setting the confidence score to the highest score 0.9, and choosing the default setting for the rest of the parameters to achieve the PPI network. The created network was exported to Cytoscape 3.9.1 software (https://www.cytoscape.org (accessed on 1 February 2023)) [54].

**Figure 6:**
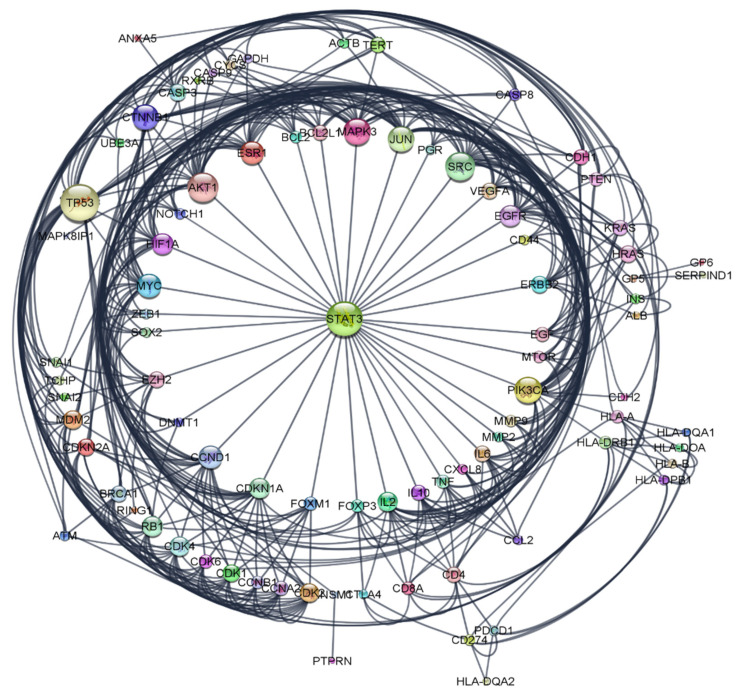
Network with radial layout, nodes represent cervical cancer protein targets, and the edges represent protein–protein interactions. The size of nodes signifies the connectivity of each protein; the higher the node size the higher its connectivity to other nodes.

The resulted network comprised of 168 nodes and 1038 edges. A filter tool was applied to exclude the nodes with degree of connectivity below the median score of 9. The nodes above these values were selected and visualized as a radial layout. This algorithm places the nodes in a concentric virtual circle around a common node center (Figure 6). The generated network consisted of 87 nodes, each node represents a specific protein and 463 edges; each edge indicates the interaction between the proteins in the constructed network [55].

#### 2.6.3. Hub Gene Expression Analysis

**Figure 7:**
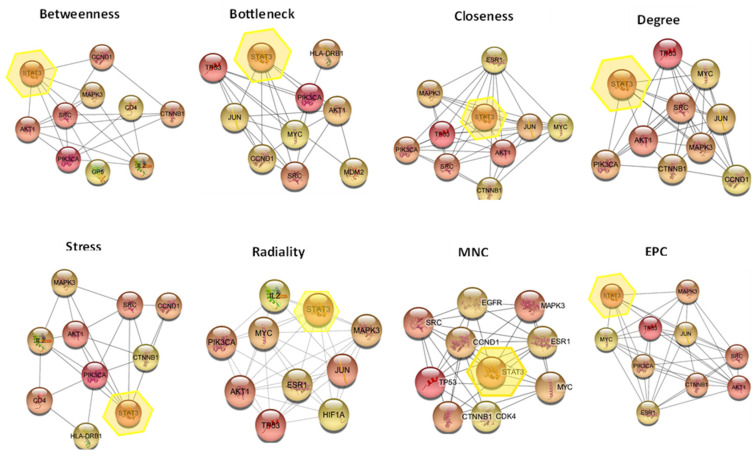
The occurrence of STAT3 in analysis methods of cytoHubba, STAT3 was present in eight methods (Betweenness Method, Bottleneck Method, Closeness Method, Degree Method, EPC Method, MNC Method, Radiality Method, Stress Method) of twelve.

The *cytoHubba* plugin Cytoscape is considered a useful exploring interface for the most important nodes in the PPI networks. It is used to determine the hub genes using ranking methods like (degree, edge percolated component (EPC), maximum neighborhood component (MNC), the density of maximum neighborhood component (DMNC), and maximal clique centrality (MCC), bottleneck, eccentricity, closeness, radiality, betweenness, and stress, clustering coefficient) [55,56]. The results shown in Table 3 demonstrated that STAT3 was found to be present in 8 of the 12 methods, followed by SRC that was present in 7 methods, while AKT1, JUN, CTNNB1, MAPK3, MYC, TP53 were present in 6 methods, ESR1 in 4 methods, IL2 in 3 methods, and HLA-DRB1, CXCL8, CD4, CDK4 were present in 2 methods. A graphical representation for the occurrence of filtered proteins in different methods of cytoHubba is shown in Figure 7. The STAT3 protein was selected for further docking.

#### 2.6.4. Gene Ontology and Enrichment Analysis

**Figure 8:**
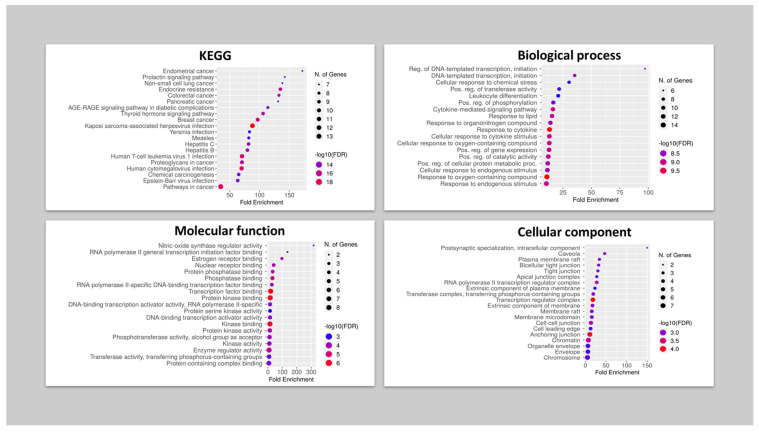
Functional enrichment analysis of filtered 16 protein coding genes by ShinyGO, including KEGG pathways, biological process, molecular function, and cellular component.

In this study, a free online tool (ShinyGO v0.76.3) was used, which represents a bioinformatic utility for performing enrichment analysis for the gene coding proteins selected, including gene ontology and pathways determination. It retrieves a comprehensive description of biological signal transduction pathways from many different databases, so the analysis was performed on the 16 genes to discover the cellular components, molecular function and biological processes that were affected by this set of genes using ShinyGO [57]. The analysis revealed that postsynaptic specialization, caveola and the plasma membrane raft were the top cellular components in the same order, while nitric oxide synthase was the top molecular function, followed by RNA polymerase initiation factor and estrogen receptor binding. For the biological processes category, screened genes were correlated with the regulation of DNA templated transcription followed by the cellular response to chemical stress. Finally, the KEGG pathway for the selected protein coding genes was found to be involved in the endometrial cancer pathway, prolactin signaling pathway and non-small cell lung cancer pathway (Figure 8).

#### 2.6.5. In Silico Molecular Docking of *Epi*-Obtusane and STAT 3 Receptor

**Figure 9:**
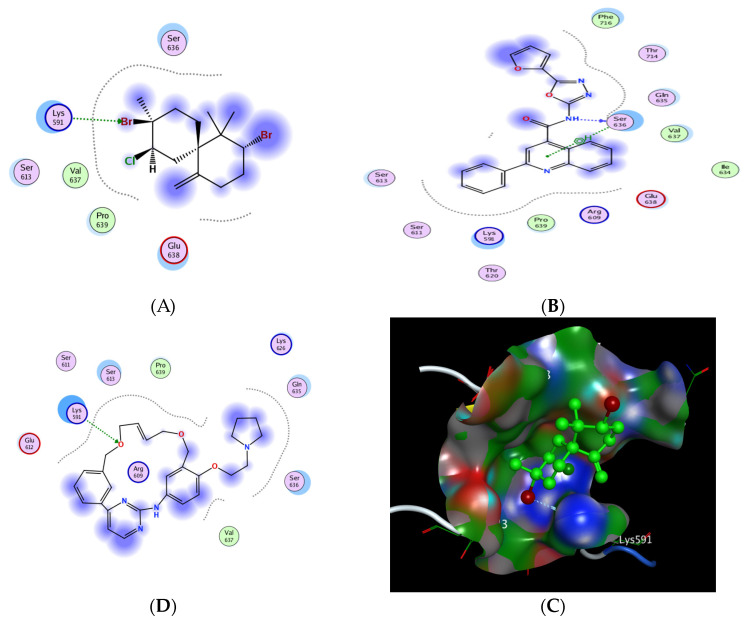
2D interactions of *epi*-obtusane (**A**), STX-0119 (**B**)Pacritinib (**C**) and 3D docking pose of *epi*-obtusane at the STAT3 SH2 domain (**D**) (PDB code:1BG1).

The signal transducer and activator of transcription 3 (STAT3) is considered one of the most important proteins in the STAT protein family due to its interference in various critical cellular processes, such as cell growth and the apoptosis process, by regulating the gene expression of the corresponding involved genes. Several studies showed that STAT3 is abnormally expressed in cervical cancer and affects tumor angiogenesis and invasion. STAT3 enters the nucleus to begin the gene regulation process and promotes the expression of genes related to the malignancy transformation, including proliferation and cell divisions [58]. The main proposed mechanism of the inhibitors of the STAT3 signaling pathway is through directly binding to the STAT3 SH2 domain and inhibiting its activation. Moreover, the SH2 domain possesses a dual function as a receptor recruitment module rather than a dimerization domain, which represents the preferable binding site of DNA with STATs. Consequently, the SH2 domain has become the favored target in the rational design and inhibition of STAT3 phosphorylation and/or dimerization, arising as one of the promising targets for developing anti-cancer motifs (Figure 9) [59,60,61,62].

The designated molecular docking was performed using “Molecular Operating Environment 2019.0102 software (MOE)”. The investigation was based on The X-ray crystallographic structure of the STAT3b homodimer bound to DNA, determined at 2.25-Å resolution (http://www.rcsb.org/pdb/ (accessed on 15 March 2023), code 1BG1). The preparation of the protein included the removal of water molecules, followed by quick preparation using the tool incorporated into the software, and then by docking the conformations stored in database for the investigational compounds using the previously synthesized oxadiazole derivative (STX- 0119)as the standard inhibitor [63,64,65].

The results showed that *epi*-obtusane achieved a comparable binding score of −3.606 Kcal/mole, while STX-0119 recorded an energy score of −5.026 Kcal/mole. Pacritinib exhibited energy score of −5.292. Regarding interactions, *epi*-obtusane showed one hydrogen bond interaction with LYS 591 as the hydrogen bond acceptor through the bromine atom attached to the cyclohexyl moiety, while Pacritinib possessed the same pattern of interaction as *epi*-obtusane, regarding the hydrogen bond interactions as the H-acceptor with LYS 591 amino acid residues (Table 4).

#### 2.6.6. Effect of *Epi*-Obtusane on STAT3 Total in HeLa Cells

It is reported that STAT3 plays a pivotal role in cancer progression. It serves as a key mediator in transmitting signals from various receptor and non-receptor tyrosine kinases that are commonly activated in cancer cells. Additionally, STAT3 acts as a transcription factor, influencing the expression of numerous genes, thereby facilitating tumor advancement. An intriguing potential avenue for cancer therapy involves targeting STAT3 inhibition, given its critical role not only in cancer cells, but also in stromal cells, including immune cells, which are recruited to the tumor microenvironment to consequently support tumor progression [66].

Semiquantitative measurement of total STAT3 and p-STAT3 in human cell lysates were performed using the in vitro ELISA assay to measure the inhibitory properties of *epi*-obtusane using Pacritinib (a novel STAT3 inhibitor) as reference; the results revealed that *epi*-obtusane recorded an inhibition to STAT3 of 6.168 ng/mL as it decreased more than double the value of the control at 13.67 ng/mL, achieving the third activity of the potent STAT3 inhibitor Pacritinib which recorded a value of 2.253 ng/mL. Furthermore, *epi*-obtusane achieved remarkable decline in the level of phosphorylated STAT3 to 19.94 ng/mL, compared with the control value of 45.24 ng/mL, while cells treated with Pacritinib showed a p-STAT3 level of 13.09 ng/mL. This effect possessed by *epi*-obtusane, may be attributed to its binding to the SH2 domain and preventing the phosphorylation of STAT3. This inhibitory effect is aligned with our findings regarding cell cycle analysis, as many studies showed that STAT3 inhibition induces G2/M arrest in cancer cells. Additionally, suppressing STAT3 signaling is of importance in triggering apoptosis (Table 5) [67].

## 3. Materials and Methods

### 3.1. Epi-Obtusane Material

In total, 5 mg of the compound *epi*-obtusane used during this work was previously isolated by Hegazy, et al. [36]. It was previously isolated from the Egyptian sea hare, *Aplysia oculifera*, which was gathered by hand at a depth of 1–1.5 m, 40 km south of Safaga City (Red Sea governorate, Egypt), in April 2011. The sample was immediately frozen. A voucher specimen of the *Aplysia* sample (06RS60) was kept at the National institute of Oceanography and Fisheries, Marine Biological Station, Hurghada, Red Sea, Egypt. Structure elucidation for the compound was carried out using spectroscopic analysis including HREIMS, ^1^H, ^13^C, DEPT, ^1^H–^1^H COSY, HMQC, and HMBC NMR; the relative configuration was confirmed by X-ray analysis.

### 3.2. Preparation of Epi-Obtusane-Containing Liposomes

Liposomes were prepared by the spraying technique [68]. Briefly, 3 mg of *epi*-obtusane was prepared via 60 mmole lipoid S75 and cholesterol (25% *w*/*w*), which were dissolved in 2 mL of absolute ethanol (organic phase). Additionally, this phase-was then sprayed (400 µL/20 s) on the surface of 9% sucrose solution with continuous 1200 rpm at 55 °C for one hour to ensure the formation of bilayer structure of liposomes. The prepared liposomal formulation was sonicated for 20 min at 25 °C, and then kept overnight at 4 °C for complete formation of the membrane.

### 3.3. Characterization of Epi-Obtusane- Containing Liposomes

Nano Zetasizer-ZSP (Malvern-Instruments, Malvern, UK) was utilized to verify the size and polydispersity index of *epi*-obtusane-containing liposomes. Liposomes were analyzed at 25 °C three times after dilution with deionized water, and the average values were determined [69]. The zeta-potential-of-the-prepared-liposomes was evaluated utilizing the Mastersizer (3000E-Malvern-Instruments, Parishes, UK) [70]. Imaging of the formulated *epi*-obtusane-containing liposomes was carried out using a scanning electron microscope (JEM-1400, Jeol, Tokyo, Japan) operated at 80 kV. Prepared liposomes were kept for ten minutes on a copper grid coated with carbon [71].

### 3.4. Antiproliferative Assay

The adenocarcinoma (HeLa) cell lines were grown in earth RPMI 1640 medium with 10% heat-inactivated fetal bovine serum (FBS) at 37 °C and 5% CO_2_. Cell lines were counted and seeded on 96-well cell culture plates (1 × 10^5^) cells). Cells were treated with certain concentrations of the test compound for 24 h in triplicates. Cell viability was determined by adding a 20µL 3-(4,5-dimethylthiazol-2-yl)-2,5-diphenyltetrazolium bromide (MTT) assay. Each plate that included untreated cells was considered 100% viable, while plates treated with a mixture of 200 ng/mL TNF, 200 ng/mL TRAIL, 200 ng/mL CD95L, 5 μg/mL CHX and 1% (*w*/*v*) sodium azide 20% were considered to have 0% viability. Optical density was determined at 560 nm and subtract background at 620 nm. The experiment was performed in triplicate and the results were calculated.

### 3.5. Cell Cycle Analysis on HeLa Cell Line Treated with Epi-Obtusane

The HeLa cells were treated with *epi*-obtusane at its 76.8 ± 2.3 µg/mL (IC_50_ concentrations for 24 h). After treatment, the cells were washed twice with ice-cold phosphate buffer saline (PBS), collected by centrifugation, and fixed in ice-cold 70% (*v*/*v*) ethanol, washed with PBS, re-suspended with 0.1 mg/mL RNase, stained with 40 mg/mL propidium iodide (PI), and analyzed by flow cytometry using FACSCalibur (Becton Dickinson, BD Biosciences, San Jose, CA, USA). The cell cycle distributions were calculated using CellQuest software V 5.1 (Becton Dickinson). Exposing HeLa cells to these compounds resulted in an interference with the normal cell cycle distribution as indicated.

### 3.6. Apoptosis Determination by Annexin-V Assay

Apoptosis was determined by flowcytometry based on the annexin V- (FITC) and propidium iodide (PI) staining kit (Bio Vision Research, Mountain View, CA 94043 USA— See Supplementary File S1).

### 3.7. In Silico Molecular Docking of Epi-Obtusane and STAT 3 Protein

Using the incorporated site finder tool in MOE, the protein active site was located via determining the classic phosphor tyrosine peptide–SH2 domain, followed by adding dummies to the alpha centers then initiating the docking tool. The dummy atoms were chosen to be the site of docking. The docking placement methodology was adjusted to be on the alpha triangle. The refinement of the post placement selected the receptor to be rigid, and the initial scoring function London dG was used and maintained to its default values. The MDB file of the investigated compounds, *epi*-obtusane, STX-0119 and Pacritinib, was prepared and loaded, then the dock calculations were automatically performed. The results were examined to explore the protein–ligand interactions at two levels of visualization, 2D and 3D. The poses were filtered according to two criteria: the energy score and the interactions between the ligand and the protein, so that the selection of poses was conducted according to their better-obtained binding scores and RMSD_Refine values. The obtained scores and interactions with the binding pocket site of the protein are discussed [65].

### 3.8. Effect of Epi-Obtusane on STAT3 and p-STAT3 Total in HeLa Cells

The protocol of the procedure, as described in the ab176655 STAT3 Total Simple Step ELISA^®^ Kit(Cambridge, CB2 0AX, UK), was summarized after preparing all reagents, samples, and controls, as instructed, in 50 µL of the control, and the sample was added to the appropriate wells after 50 μL of the antibody cocktail was added to all wells. Then, the sample was incubated for 1 h at room temperature. After, each well was washed three times with 350 μL 1X Wash Buffer PT, followed by adding 100 μL of the TMB substrate to each well and incubated for 15 min. Finally, 100 μL of the stop solution was added, and the OD was measured at 450 nm considering the p-STAT3 procedure described in ab279941—Phosphotyrosine STAT3 ELISA Kit guide (See Supplementary File S2).

## 4. Conclusions

In conclusion, *epi*-obtusane’s potential biological activity was assessed virtually using the PASS software tool, indicating its antiproliferative activity towards cervical cancer. The sesquiterpene metabolite was investigated in vitro for its anticancer potential against the HeLa cell line, as it showed moderate antiproliferative potential with an IC_50_ value of 76.8 µg/mL. The enhanced endorsement by the studied cell line was attained by the encapsulation of the metabolite within liposomes formulation. The results showed the influence of entrapment of *epi*-obtusane as a promising approach to improve the anti-proliferative potential of the metabolite (IC_50_ 15.65 µg/mL). Subsequently, a network pharmacology analysis, which focused on target genes, identified STAT3 as a key therapeutic target for cervical cancer. Furthermore, GO and KEGG analysis of the screened protein coding genes revealed that the enriched function and pathway were primarily related to postsynaptic specialization, nitric oxide synthase, the endometrial cancer pathway and the regulation of DNA templated transcription. The docking study results on the STAT3 SH2 domain revealed that *epi*-obtusane acquired an acceptable binging score when compared with the standard inhibitor (STX- 0119). The results reveal the impact of the entrapment *epi*-obtusane as a promising approach to enhance the anti-proliferative potential of the metabolite. However, further in vivo studies should be implemented to confirm that the concept may lead to the development of a new anticancer drug for cervical cancer.

## Figures and Tables

**Table 1:** Cell cycle analysis on HeLa cell line treated with *epi*-obtusane.

No.	Sample Data	DNA Content			
	Code	%G0-G1	%S	%G2/M	Comment
**1**	*Epi*-obtusane/HeLa	46.29	28.72	24.99	cell growth arrest @ G2/M phase
**2**	Cont. HeLa	54.62	32.86	12.52	

**Table 2:** Percentage of apoptosis and necrosis for *epi*-obtusane on HeLa cells.

Compound		Apoptosis			Necrosis
		Total	Early	Late	
**1**	*Epi*-obtusane/HeLa	39.61	22.24	12.51	4.86
**2**	Control	1.84	0.37	0.18	1.29

**Table 3:** List of the protein coding genes present in at least two methods from twelve different methods of the cytoHubba plugin Cytoscape.

Name	Occurrence
STAT3	8
SRC	7
PIK3CA	6
AKT1	6
JUN	6
CTNNB1	6
MAPK3	6
MYC	6
TP53	6
CCND1	6
ESR1	4
il2	3
HLA-DRB1	2
CXCL8	2
CD4	2
CDK4	2

**Table 4:** Receptor interactions and binding energies of the *epi*-obtusane, STX-0119 and Pacritinib at the active site of STAT 3 (PDB code:1BG1).

No.	Compound	S ^a^ kcal/mole	Amino Acid Bond	Distance Å	E (Kcal Mol)
**1**	*Epi*-obtusane	−3.606	Lys 591/H-acceptor	3.1	−1.3
**2**	STX-0119	−5.026	Ser 636/H-donor	2.9	−6.1
			Ser 636/pi H	4.43	−0.8
**3**	Pacritinib	−4.991	Lys 591/H-acceptor	3.3	−1.7

^a^ S: the score of a compound placement inside the protein binding pocket.

**Table 5:** Effect of *epi*-obtusane and Pacritinib on the level of STAT3 and p-STAT3 in HeLa cells.

No.	Code	STAT3 ng/mL	nM	Folds	p-STAT3 ng/mL	nM	Folds
**1**	*Epi*-Obtusane/HeLa	6.168 ± 0.31	15.4	0.45	19.94 ± 0.37	50.35	0.44
**2**	Pacritinib/HeLa	2.253 ± 0.04	4.7	0.16	13.09 ± 0.67	27.69	0.28
**3**	Control	13.67 ± 1.01		1	45.24 ± 2.03		1

## Data Availability

Not applicable.

## Supplementary Materials

The following supporting information can be downloaded at: https://www.mdpi.com/article/10.3390/ph16111578/s1, File S1: annexin V- (FITC) and propidium iodide (PI) staining kit Bio Vision Research, Mountain View, CA 94043 USA; File S2: ab279941 – Phosphotyrosine STAT3 ELISA Kit guide.
