# Supplementary material for: Pro-Apoptotic Activity of Epi-Obtusane against Cervical Cancer: Nano Formulation, In Silico Molecular Docking, and Pharmacological Network Analysis
Source: Pharmaceuticals (Basel). 2023 Nov 8;16(11):1578. doi: 10.3390/ph16111578 (PMC10674245; doi:10.3390/ph16111578)
Supplement: Supplementary file 1 [file pharmaceuticals-16-01578-s001.zip › File S1.pdf]

BioVision rev. 04/11

For research use only

Annexin V-FITC Apoptosis Detection Kit

(Catalog #: K101-25, -100, -400; Store at 4°C; Stable for one year)

I. Introduction:

Annexin V Apoptosis Detection Kit is based on the observation that soon after initiating apoptosis, cells translocate the membrane phosphatidylserine (PS) from the inner face of the plasma membrane to the cell surface. Once on the cell surface, PS can be easily detected by staining with a fluorescent conjugate of Annexin V, a protein that has a high affinity for PS. The one-step staining procedure takes only 10 minutes. Detection can be analyzed by flow cytometry or by fluorescence microscopy. The kit can differentiate between apoptosis and necrosis when performing both Annexin V-FITC and PI staining.

II. Kit Contents:

Components	K101-25	K101-100	K101-400	Part Number
	25 assays	100 assays	400 assays	
Annexin V-FITC	125 µl	500 µl	2 ml	K101-XX(X)-1
1X Binding Buffer	12.5 ml	50 ml	2 x 100 ml	K101-XX(X)-2
Propidium Iodide (PI)	125 µl	500 µl	2 ml	K101-XX(X)-3

III. Annexin V-FITC Assay Protocol:

A. Incubation of cells with Annexin V-FITC

1. Induce apoptosis by desired method.
2. Collect 1-5 x 10^5 cells by centrifugation.
3. Resuspend cells in 500 µl of 1X Binding Buffer.
4. Add 5 µl of Annexin V-FITC and 5 µl of propidium iodide (PI 50mg/ml, optional.)
5. Incubate at room temperature for 5 min in the dark.

Proceed to B or C below depending on method of analysis.

B. Quantification by Flow Cytometry

Analyze Annexin V-FITC binding by flow cytometry (Ex = 488 nm; Em = 530 nm) using FITC signal detector (usually FL1) and PI staining by the phycoerythrin emission signal detector (usually FL2).

For adherent cells, gently trypsinize and wash cells once with serum-containing media before incubation with Annexin V-FITC (A.3-5).

C. Detection by Fluorescence Microscopy

1. Place the cell suspension from Step A.5 on a glass slide. Cover the cells with a glass coverslip.

For analyzing adherent cells, grow cells directly on a coverslip. Following incubation (A.5), invert coverslip on a glass slide and visualize cells. The cells can also be washed and fixed in 2% formaldehyde before visualization. (Cells must be incubated with Annexin V-FITC before fixation since any cell membrane disruption can cause nonspecific binding of Annexin V to PS on the inner surface of the cell membrane.)

2. Observe the cells under a fluorescence microscope using a dual filter set for FITC & rhodamine.

Cells that have bound Annexin V-FITC will show green staining in the plasma membrane. Cells that have lost membrane integrity will show red staining (PI) throughout the nucleus and a halo of green staining (FITC) on the cell surface (plasma membrane).

RELATED PRODUCTS:

Apoptosis Detection Kits & Reagents
- Annexin V Kits & Bulk Reagents
- Caspase Assay Kits & Reagents
- Mitochondrial Apoptosis Kits & Reagents
- Nuclear Apoptosis Kits & Reagents
- Apoptosis Inducers and Set
- Apoptosis siRNA Vectors

Cell Fractionation System
- Mitochondria/Cytosol Fractionation Kit
- Nuclear/Cytosol Fractionation Kit
- Membrane Protein Extraction Kit
- Cytosol/Particulate Rapid Separation Kit
- Mammalian Cell Extraction Kit
- FractionPREP Fractionation System

Cell Proliferation & Senescence
- Quick Cell Proliferation Assay Kit
- Senescence Detection Kit
- High Throughput Apoptosis/Cell Viability Assay Kits
- LDH-Cytotoxicity Assay Kit
- Bioluminescence Cytotoxicity Assay Kit
- Live/Dead Cell Staining Kit

Cell Damage & Repair
- HDAC & HAT Fluorometric & Colorimetric Assays & Drug Discovery Kits
- DNA Damage Quantification Kit
- Glutathione & Nitric Oxide Fluorometric & Colorimetric Assay Kits

Signal Transduction
- cAMP & cGMP Assay Kits
- Akt & JNK Activity Assay Kits
- Beta-Secretase Activity Assay Kit

Adipocyte & Lipid Transfer
- Recombinant Adiponectin, Survivin, & Leptin
- CETP & PLTP Activity Assay & Drug Discovery Kits
- Total Cholesterol Quantification Kit

Molecular Biology & Reporter Assays
- siRNA Vectors
- Cloning Insert Quick Screening Kit
- Mitochondrial & Genomic DNA Isolation Kits
- 5 Minutes DNA Ligation Kit
- 20 Minutes Gel Staining/Destaining Kit
- b-Galactosidase Staining Kit & Luciferase Reporter Assay Kit

Growth Factors and Cytokines

BioVisionResearch Products
980 Linda Vista Avenue, Mountain View, CA 94043 USA

Tel: 650-428-0236 · Fax: 650-428-0336 · www.biovision.com
Email: tech@biovision.com

GENERAL TROUBLESHOOTING GUIDE FOR ANNEXIN BASED KITS:

Problems	Cause	Solution
**High Background**	• Cell density is higher than recommended<br>• Increased volumes of components added<br>• Incubation of cell samples for extended periods<br>• Use of extremely confluent cells<br>• Contaminated cells	• Refer to datasheet and use the suggested cell number<br>• Use calibrated pipettes accurately<br>• Refer to datasheets and incubate for exact times<br>• Perform assay when cells are at 80-95% confluency<br>• Check for bacteria/ yeast/ mycoplasma contamination
**Lower signal levels**	• Washing cells with PBS before/after fixation (adherent cells)<br>• Cells did not initiate apoptosis<br>• Very few cells used for analysis<br>• Incorrect setting of the equipment used to read samples<br>• Use of expired kit or improperly stored reagents	• Always use binding buffer for washing cells<br>• Determine the time-point for initiation of apoptosis after induction (time-course experiment)<br>• Refer to data sheet for appropriate cell number<br>• Refer to datasheet and use the recommended filter setting<br>• Always check the expiry date and store the components appropriately
**Erratic results**	• Uneven number of cells seeded in the wells<br>• Adherent cells dislodged at the time of experiment<br>• Incorrect incubation times or temperatures<br>• Incorrect volumes used<br>• Increased or random staining observed in adherent cells	• Seed only healthy cells (correct passage number)<br>• Perform experiment gently and in duplicates or triplicates for each treatment<br>• Refer to datasheet & verify correct incubation times and temperatures<br>• Use calibrated pipettes and aliquot correctly<br>• Always stain cells with Annexin before fixation (makes cell membrane leaky)
**Note#** The most probable cause is listed under each section. Causes may overlap with other sections.		

BioVisionResearch Products
980 Linda Vista Avenue, Mountain View, CA 94043 USA

Tel: 650-428-0236 · Fax: 650-428-0336 · www.biovision.com
Email: tech@biovision.com
