# Supplementary material for: Pro-Apoptotic Activity of Epi-Obtusane against Cervical Cancer: Nano Formulation, In Silico Molecular Docking, and Pharmacological Network Analysis
Source: Pharmaceuticals (Basel). 2023 Nov 8;16(11):1578. doi: 10.3390/ph16111578 (PMC10674245; doi:10.3390/ph16111578)
Supplement: Supplementary file 1 [file pharmaceuticals-16-01578-s001.zip › File S2.pdf]

ab279941 – Phosphotyrosine STAT3 ELISA Kit

Phosphotyrosine STAT3 ELISA Kit (ab279941) is a very rapid, convenient and sensitive assay kit that can monitor the activation or function of important biological pathways in human cell lysates. By determining phosphorylated STAT3 protein in your experimental model system, you can verify pathway activation in your cell lysates. You can simultaneously measure numerous different cell lysates without spending excess time and effort in performing a Western Blotting analysis.

This Sandwich ELISA kit is an *in vitro* enzyme-linked immunosorbent assay for the measurement of human and mouse phospho-STAT3. An anti-STAT3 antibody has been coated onto a 96-well plate. Samples are pipetted into the wells and phosphorylated and unphosphorylated STAT3 present in a sample is bound to the wells by the immobilized antibody. The wells are washed and biotinylated anti-phosphotyrosine antibody is used to detect only tyrosine-phosphorylated protein. After washing away unbound antibody, HRP-conjugated streptavidin is pipetted to the wells. The wells are again washed, a TMB substrate solution is added to the wells and color develops in proportion to the amount of phospho-STAT3 bound. The Stop Solution changes the color from blue to yellow, and the intensity of the color is measured at 450 nm.

For research use only - not intended for diagnostic use.

For overview, typical data and additional information please visit: www.abcam.com/ab279941

Storage and Stability

The entire ELISA kit may be stored at -20°C for up to 6 months from the date of shipment. Avoid repeated freeze-thaw cycles. For extended storage, it is recommended to store at -80°C.

Materials Supplied

Item	Quantity	Storage Condition
Pan STAT3 Coated Microplate	1 unit	-20°C
20X Wash Buffer Concentrate	25 mL	-20°C
Positive Control-treated A431 cell lysate	1 vial	-20°C
Biotinylated anti-phosphotyrosine antibody	2 vials	-20°C
2000X HRP-conjugated anti-rabbit IgG concentrate	1 vial	-20°C
TMB One-Step Substrate Reagent	12 mL	-20°C
Stop Solution	8 mL	-20°C
5X Assay Diluent	15 mL	-20°C
2X Cell Lysate Buffer	10 mL	-20°C

Materials Required, Not Supplied

These materials are not included in the kit, but will be required to successfully utilize this assay:

- Microplate reader capable of measuring absorbance at 450 nm.
- Protease and Phosphatase inhibitors.
- Precision pipettes to deliver 2 µl to 1 ml volumes.
- Adjustable 1-25 ml pipettes for reagent preparation.
- 100 ml and 1 litre graduated cylinders.
- Distilled or deionized water.
- Log-log graph paper or computer and software for ELISA data analysis.
- Tubes to prepare the positive control or sample dilutions.

Cell Lysate Preparation

Rinse the cells with PBS, making sure to remove any remaining PBS before adding the lysis buffer. Solubilize cells at 4 x 10^7 cells/ml in prepared Cell Lysate Buffer (see Reagent Preparation section). Pipette up and down to resuspend the pellet. Incubate the lysates with shaking at 2-8°C for 30 minutes. Microcentrifuge at 13,000 rpm for 10 mins at 2-8°C and transfer the supernatants into a clean test tube. Lysates should be used immediately or aliquoted and stored at -70°C. Avoid repeated freeze-thaw cycles. Thawed lysates should be kept on ice prior to use.

For the initial experiment, we recommend a serial dilution, such as a 5-fold to 100-fold dilution, for your cell lysates with prepared Assay Diluent (see Reagent Preparation section) before use.

Δ Note: *The fold dilution of sample used depends on the abundance of phosphorylated proteins and should be determined empirically. More of the sample can be used if signals are too weak. If signals are too strong, the sample can be diluted further.*

Positive Control Preparation

Briefly spin the Positive Control Vial. Add 400 µl of prepared 1X Assay Diluent into the vial to prepare a Positive Control (P-1) solution. Gently mix the powder to allow it to dissolve thoroughly. If a precipitate is seen in the solution after mixing, this can be removed by a quick centrifuge of the positive control vial, and then pipetting the supernatant only for the assay. Pipette 300 µl 1X Assay Diluent into a further four tubes, label them (P-2) to (P-5). Use the Positive Control (P-1) solution to produce a dilution series, pipetting 150 µl from (P-1) into (P-2), and so on. Mix each tube thoroughly before the next transfer. 1X Assay Diluent serves as the blank (P-0).

Reagent Preparation

5X Assay Diluent:
Dilute 5X Assay Diluent 5-fold with deionized or distilled water before use.
Cell Lysate Buffer:
Cell lysate buffer should be diluted 2-fold with deionized or distilled water (for cell lysate and tissue lysate). We also recommend the addition of protease and phosphatase inhibitors (not included) to the lysis buffer prior to use.
Biotinylated anti-phosphotyrosine antibody:
Briefly spin the vial. Add 100 µl of 1X Assay Diluent into the vial to prepare a phospho detection antibody concentrate. Pipette up and down to mix gently (the concentrate can be stored at 4°C for 5 days or at -80°C for one month). The concentrate should then be diluted 80- fold with 1X Assay Diluent and used in step 5 of the Assay Procedure.
20X Wash Buffer:
If the Wash Concentrate (20X) contains visible crystals, warm to room temperature and mix gently until dissolved. Dilute 20 mL of Wash Buffer Concentrate into deionized or distilled water to yield 400 mL of 1X Wash Buffer.
HRP-conjugated Streptavidin concentrate:
HRP-conjugated Streptavidin should be diluted 600-fold with 1X Assay Diluent and used in step 7 of the Assay Procedure.
For example: Briefly spin the vial. Add 20 µl of HRP-conjugated Streptavidin concentrate into a tube with 12 mL 1x Assay Diluent, pipette up and down to mix gently to prepare a 600-fold solution. Mix well.

Assay Procedure

1. Bring all reagents and samples to room temperature (18 - 25°C) before use. It is strongly recommended to run all positive controls and samples in at least duplicate.
2. Label removable 8-well strips as appropriate for your experiment.
3. Add 100 µl of positive control or sample into appropriate wells. Cover the wells and incubate for 2.5 hours at room temperature or overnight at 4°C with gentle shaking.
4. Discard the solution and wash 4 times with 1X Wash Solution. Wash by filling each well with Wash Buffer (300 µl) using a multi-channel Pipette or auto washer. Complete removal of liquid at each step is essential for good performance. After the last wash, remove any remaining Wash Buffer by aspirating or decanting. Invert the plate and blot it against clean paper towels.
5. Add 100 µl of prepared 1X biotinylated anti-phosphotyrosine antibody to each well. Incubate for 1 hour at room temperature with gentle shaking.
6. Discard the solution. Repeat the wash as in step 4.
7. Add 100 µl of prepared HRP-conjugated Streptavidin solution to each well. Incubate for 45 minutes at room temperature with gentle shaking.
8. Discard the solution. Repeat the wash as in step 4.
9. Add 100 µl of TMB One-Step Substrate Reagent to each well. Incubate for 30 minutes at room temperature in the dark with gentle shaking.
10. Add 50 µl of Stop Solution to each well. Read at 450 nm immediately.
11. Calculate the mean absorbance for each set of duplicate positive controls and samples, and then subtract the average zero (blank) optical density.

Download our ELISA guide for technical hints, results, calculation, and troubleshooting tips:
www.abcam.com/protocols/the-complete-elisa-guide

For technical support contact information, visit: www.abcam.com/contactus

Copyright © 2021 Abcam, All Rights Reserved. All information / detail is correct at time of going to print.
Version 1 | 2021-Jan-15
